# Salivary Cortisol as a Biomarker of Sleep Quality: A Scoping Review

**DOI:** 10.3390/clockssleep8040068

**Published:** 2026-10-20

**Authors:** Preyahathai Aroonvanichporn, Poompat Thititanakul, Krittapak Kwanmongkolpong, Nicharat Sriratanasak, Chanchai Boonla, Chidsanu Changsiripun, Nattida Chotechuang

**Affiliations:** 1 NIST International School, Bangkok 10110, Thailand; homey2551@gmail.com; 2 Department of Food Technology, Faculty of Science, Chulalongkorn University, Bangkok 10330, Thailand; 6532070423@student.chula.ac.th (P.T.); 6970003023@student.chula.ac.th (K.K.); 3 Department of Pharmacology and Physiology, Faculty of Pharmaceutical Sciences, Chulalongkorn University, Bangkok 10330, Thailand; nicharat.sri@outlook.com; 4 Center of Excellence in Cancer Cell and Molecular Biology, Faculty of Pharmaceutical Sciences, Chulalongkorn University, Bangkok 10330, Thailand; 5 Department of Biochemistry, Faculty of Medicine, Chulalongkorn University, Bangkok 10330, Thailand; chanchai.b@chula.ac.th; 6 Department of Orthodontics, Faculty of Dentistry, Chulalongkorn University, Bangkok 10330, Thailand; chidsanu.c@chula.ac.th

**Keywords:** cortisol awakening response, diurnal cortisol, non-invasive method, sleep measurement, hypothalamic–pituitary–adrenal axis (HPA axis)

## Abstract

Background: Sleep is a crucial regulator for physiological and psychological balance, and sleep disturbances have been associated with the hypothalamic–pituitary–adrenal (HPA) axis. Cortisol exhibits a circadian rhythm and plays a central role in stress response and metabolic regulation. Salivary cortisol has been widely used as a non-invasive biomarker for sleep quality, but its reflection remains unclear. This scoping review aims to systematically map and evaluate the existing literature on the relationship between salivary cortisol and sleep outcomes. Methods: Medline, Scopus, and Cochrane databases were applied to identify relevant studies. Database searches combined specific terms from three concept groups: cortisol, sleep quality, and biomarkers. This protocol follows the JBI guidelines. To be incorporated, records pertaining to any type of study design that mentions the use of salivary cortisol and the analysis of sleep quality were considered. Finally, 1067 studies were imported. Among those, 31 studies met eligibility criteria and were extracted. Result: The results indicated the trend in the use of salivary cortisol as a sleep quality biomarker; the study shows limited consistency as a biomarker. Its relationship with sleep outcomes is highly dependent on methodological factors and contextual influences. Conclusions: Overall, salivary cortisol may reflect broader stress-related physiological processes rather than sleep quality directly.

## 1. Introduction

Sleep is an important physiological activity for maintaining homeostasis in the human body, as it involves many important functions like emotion regulation, metabolic processes, and immune system integrity. Many non-communicable diseases (NCDs), which include commonly present diseases like cardiovascular disease, depression, metabolic disorders, and impaired cognitive performance, have been associated with poor sleep quality [1,2,3]. To measure the sleep quality, researchers often measure physiological signals by using devices such as polysomnography and actigraphy paired with subjective questionnaires [4]. However, in clinical studies, there can be some limitations to this approach as devices for measuring physiological signals can be costly and hard to find. Thus, some studies aim to find correlations between biomarkers and sleep quality, which does not involve invasive methods like blood sampling, and by identifying the current rise in sleep quality research, such as using salivary cortisol as biomarkers [5].

**Figure 1:**
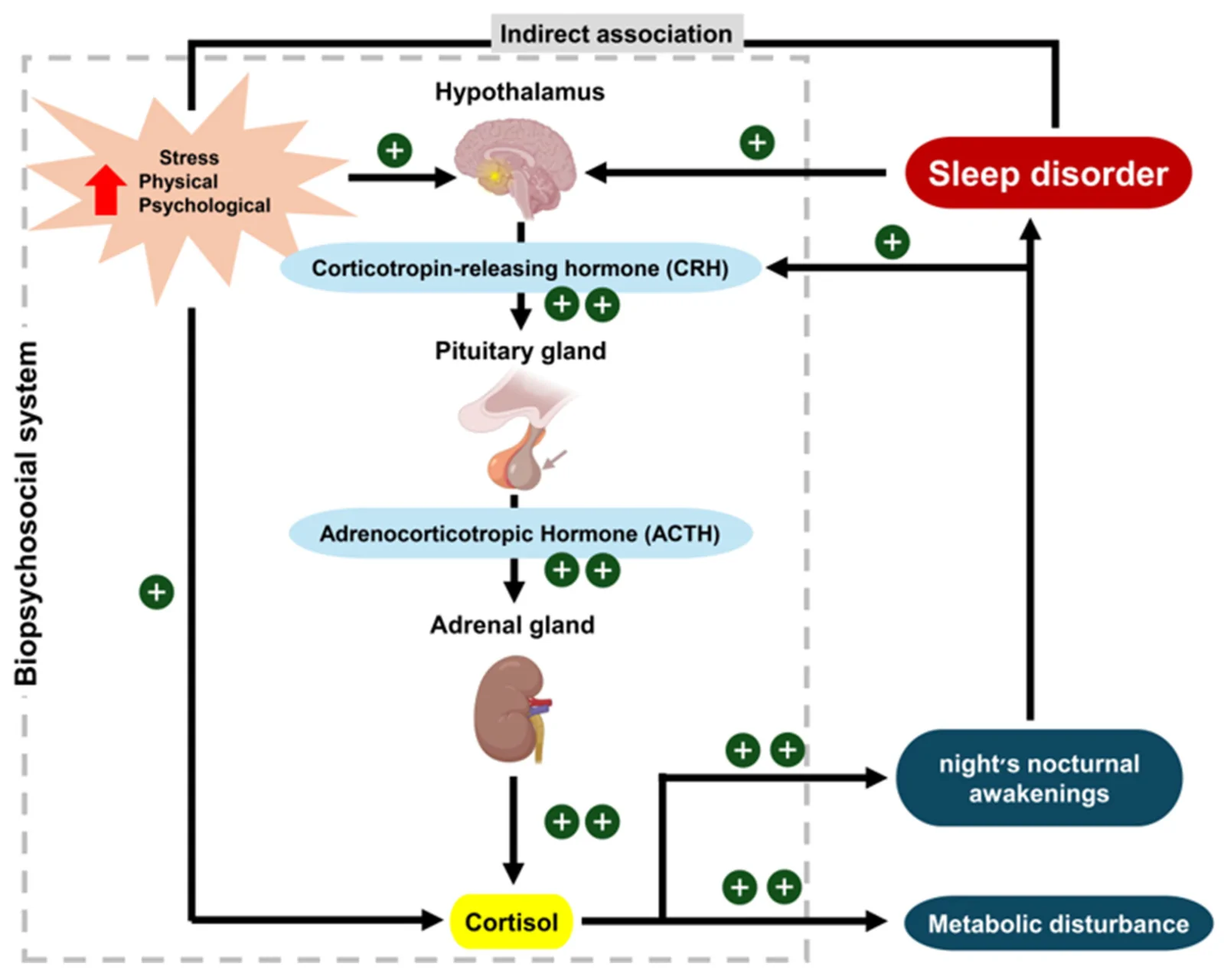
Pathway linking between salivary cortisol and sleep quality within a biopsychosocial framework. Physical and psychological stressors activate the hypothalamus, triggering corticotropin-releasing hormone (CRH) secretion, adrenocorticotropic hormone (ACTH) release from the pituitary, and subsequent cortisol production by the adrenal cortex. Elevated cortisol contributes to nocturnal awakenings and metabolic dysregulation, promoting sleep disturbance. Sleep disturbance, in turn, reactivates the hypothalamic–pituitary–adrenal (HPA) axis, creating a bidirectional reinforcing cycle. The dashed box denotes the indirect association pathway. Green (+) symbols indicate stimulatory relationships.

Cortisol is a product from the HPA axis, a primary system involved in sleep regulation and stress, which has a distinct circadian rhythm characterized by a rapid increase following awakening and a gradual decline throughout the day, also known as cortisol awakening response or CAR for short [3,6]. To provide an overview of the mechanisms linking sleep quality and salivary cortisol, Figure 1 presents a conceptual framework summarizing the interaction between psychosocial stress, sleep disturbance, and hypothalamic–pituitary–adrenal (HPA) axis activity. Physical and psychological stressors activate the hypothalamus, triggering corticotropin-releasing hormone (CRH) secretion, adrenocorticotropic hormone (ACTH) release from the pituitary, and subsequent cortisol production by the adrenal cortex. Increased cortisol contributes to nocturnal awakenings and metabolic dysregulation, thereby promoting sleep disturbance. These mechanisms make cortisol a potentially valuable indicator of physiological regulation.

There are many available biological samples that can be used to find cortisol. However, one of the most common biological samples for cortisol is serum cortisol, which involves drawing blood from the subject. An alternative and non-invasive biological sample is salivary cortisol. Salivary cortisol is considerably easier to obtain due to its non-invasive nature, and it can measure free and biologically active cortisol [7,8]. Its use allows for repeated sampling in real-world settings, which is particularly advantageous for sleep research. However, despite its widespread use, there is no consensus on how salivary cortisol should be applied to assess sleep quality [9]. But, as outlined in the protocol, existing studies that involve saliva sampling are widely varied in factors that could impact the cortisol level result, such as saliva sample storing condition, sampling time, and assay for measuring cortisol, which impacts the consistency of findings and limits the validity of salivary cortisol as a biomarker of sleep [1,10]. While previous reviews have examined salivary biomarkers in relation to sleep alterations [11] or cortisol in specific populations such as shift workers, no scoping review has comprehensively synthesized evidence on salivary cortisol as a biomarker of sleep quality across diverse populations and clinical contexts. Furthermore, the literature in this area has grown substantially in recent years.

Currently, there are many studies focusing on the relationship between sleep and physiological biomarkers, stress-related neuroendocrine markers, as well as circadian rhythm regulation [12]. However, simultaneously, such studies often only narrow down to focus on specific small populations, individual sleep disorders, or broader biomarker panels. These reviews do not incorporate evaluating salivary cortisol as a biomarker of sleep quality [11]. Additionally, evidence gathered in these studies was collected before the recent increase in studies on salivary cortisol across diverse populations and countries [13]. As of 2012, there has been a substantial increase in research on salivary cortisol compared to sleep outcomes among healthy individuals, clinical populations, adolescents, older adults, and individuals with chronic diseases. However, even so, the findings of these studies remain very inconsistent [12]. Specifically, some studies indicate how there are clear relationships between sleep quality and cortisol levels, whereas certain studies show weak, absent, or context-dependent relationships.

Previous systematic reviews have examined the use of salivary cortisol in different research contexts. One review discussed its application in randomized controlled trials (RCTs) as a measure of physiological responses to interventions [13], while another examined its use in biobehavioral research as an outcome, predictor, or descriptive measure across various domains, including stress and other behavioral and physiological outcomes [14]. Although these reviews provide an overview of the broader applications of salivary cortisol, they do not specifically address the evidence concerning its association with sleep quality as the primary focus. A focused examination of this relationship requires further examination, particularly given the differences in study populations, sleep assessment methods, cortisol sampling protocols, and outcome measures across studies.

Therefore, the objectives of this scoping review are to systematically inquire into the existing literature on salivary cortisol and sleep quality, evaluate methodological approaches, and identify limitations and gaps in knowledge to inform future research. Specifically, this review seeks to investigate populations and study designs used in the current literature, summarize prevalent methodologies used to assess sleep quality and salivary cortisol as well as identify key relationships and methodological gaps to inform further research and clinical applications.

## 2. Methods

Reviewers conducted this scoping review in accordance with the Joanna Briggs Institute (JBI) methodology for scoping reviews [15] and PRISMA Extension for Scoping Reviews (PRISMA-ScR) reporting framework [16].

We first formulated an extensive research question, which is *“*What evidence exists regarding the use of salivary cortisol as a biomarker of sleep quality across different populations and settings?*”* Then, a review protocol was developed beforehand to guide the methodological approach, including the definition of eligibility criteria, search strategy, study selection process, and data extraction framework.

The protocol is available on OSF (https://osf.io/su2d9/overview, accessed on 27 January 2026) and was used to ensure transparency, reproducibility, and methodological consistency throughout the review process.

### 2.1. Inclusion Criteria

The central concept of this review was the use of salivary cortisol as a biomarker of sleep quality. Studies were included if they examined salivary cortisol in relation to sleep quality, sleep disturbances, or related sleep variables. The research needs to have an outcome, which can be both subjective and objective assessments of sleep, such as PSQI, questionnaires, sleep diaries, actigraphy result, and polysomnography result [1,3]. This study also includes a broad range of study designs involving participants of any age, sex, and ethnicity. Additionally, no restrictions were imposed on the language of publication or participant characteristics, including health status and ethnicity.

The review also considered variations in salivary cortisol measurement, including sampling timing, frequency, cortisol awakening response, diurnal patterns, analytical methods, and interpretative frameworks [7,17].

Review articles were excluded, and studies were excluded if cortisol was measured using invasive methods such as blood sampling, if outcomes were not related to sleep quality or sleep assessment, if the studies are done in in vitro or in animal, or if the study did not directly investigate the relationship between salivary cortisol and sleep-related outcomes or had no outcome.

### 2.2. Search Strategy

A systematic search was conducted using databases including Scopus, Medline/PubMed, and CENTRAL as the main sources from their inception to 3 March 2026. Search terms combine keywords related to “cortisol”, “salivary biomarkers”, and “sleep quality” used considering the title, key and abstract fields (for a detailed search query for each database, see Table 1). There were no language restrictions in this search. Gray literature and study protocol were not considered for the study purpose. Reference lists of included studies were also screened.

Records were imported into Covidence (covidence.org) for screening. In the first step, duplicates were detected and deleted by Covidence and reviewers. In the second and third phase, titles and abstracts were screened and recorded by P.A. and P.T., followed by full-text review. Disagreements were resolved through discussion to reach consensus, and K.K. made the final decision.

### 2.3. Data Extraction

The information extracted for each record included: author(s); year of publication; country of origin; aims/purpose of the study; population; study design; type of sleep measurement; sampling cortisol; sampling timing; association between salivary cortisol and sleep quality and key findings. Details are described below:

- Author(s): author or authors who conducted the study;
- Year of publication: year in which the document/record was published;
- Country: country associated with the corresponding author of the publication;
- Aim(s) of the study: overall aim(s) and main objective(s) of the publication;
- Population: type of sample recruited for the study;
- Study design: cross-sectional, longitudinal, randomized controlled trial, non-randomized controlled trial, cohort study, etc.;
- Sleep measurement: subjective or objective assessment or both, and which tools were used to collect the data;
- Cortisol sampling: collection method, storage condition, timing, frequency, and cortisol detection assay;
- Key findings: correlation between sleep outcome and cortisol level.

### 2.4. Analysis and Visualization

A data chart was developed to extract the necessary information from included records. The figure was generated by N.S., and all content was validated by C.C. and N.C. Absolute, and relative frequencies calculation and graph were performed by GraphPad Prism 11.0.2.

## 3. Results and Discussion

### 3.1. Search Results

**Figure 2:**
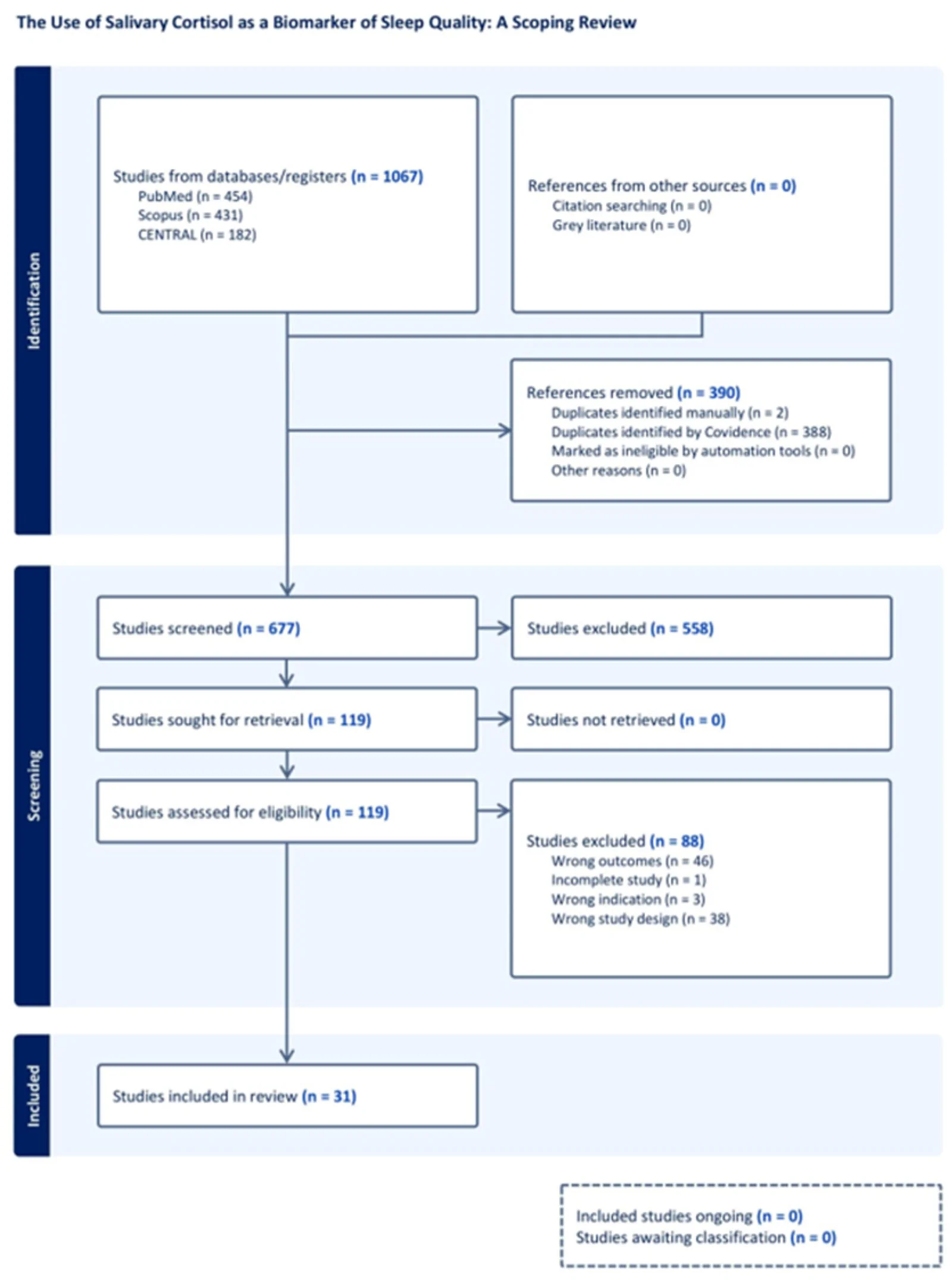
PRISMA flow diagram illustrating the study selection process. A total of 1067 records were identified from three databases (PubMed, *n* = 454; Scopus, *n* = 431; CENTRAL, *n* = 182), with no additional records identified from other sources. Following removal of 390 duplicates, 677 records were screened, of which 558 were excluded. Of the 119 studies sought, retrieved and assessed for eligibility, 88 were excluded due to wrong outcomes (*n* = 46), wrong study design (*n* = 38), wrong indication (*n* = 3), and incomplete study (*n* = 1). A final total of 31 studies were included in the review. No studies were ongoing or awaiting classification.

Figure 2 provides the PRISMA flowchart of the study. The database search yielded 1067 records across PubMed (*n* = 454), Scopus (*n* = 431), and CENTRAL (*n* = 182). After removal of 390 duplicates, 677 records remained for title and abstract screening. Of these, 558 were excluded for not meeting the inclusion criteria. A total of 119 full-text articles were assessed for eligibility, with 31 studies remaining meeting the final inclusion criteria.

#### Results of Individual Sources of Evidence

Detailed study characteristics and findings are presented in Table 2.

### 3.2. Characteristics of Sources of Evidence

The 31 included studies encompassed a wide range of methodological approaches and populations, reflecting the breadth of research on salivary cortisol and sleep. Study designs included randomized controlled trials, prospective cohort studies, cross-sectional analyses, and intensive longitudinal designs such as daily diary studies.

Sample sizes varied substantially, ranging from small experimental samples of fewer than 20 participants to large cohort-based studies exceeding 400 individuals. Populations included healthy adults, adolescents, clinical populations such as individuals with insomnia, obstructive sleep apnea, and chronic illness, as well as high-stress groups including caregivers and occupational cohorts.

Sleep outcomes were assessed using three primary approaches: subjective measures, objective measures, and combined approaches. Subjective measures, including the Pittsburgh Sleep Quality Index (PSQI), Insomnia Severity Index (ISI), and sleep diaries, were the most frequently used, employed in 16 of the 31 included studies. Objective measures such as actigraphy and polysomnography were less frequently employed but provided more physiologically grounded data, appearing in seven studies. Finally, eight studies combined both subjective and objective assessments, offering a more comprehensive evaluation of sleep.

Salivary cortisol measurement protocols varied considerably across the 31 included studies. Single-time-point sampling was employed in 6 studies, cortisol awakening response (CAR) protocols were used in 12 studies, and multi-time-point diurnal sampling was conducted in 13 studies. Analytical methods were predominantly immunoassay-based, with enzyme-linked immunosorbent assay (ELISA) being the most reported technique.

### 3.3. Synthesis of Results

Included studies were processed according to key methodological and conceptual characteristics, including sleep assessment type, cortisol sampling protocol, study design, and population characteristics. This structured approach allowed for systematic comparison across heterogeneous evidence and enabled identification of patterns influencing the relationship between salivary cortisol and sleep outcomes. The characteristics of the included studies are depicted in Methodology Section (Table 1).

**Figure 3:**
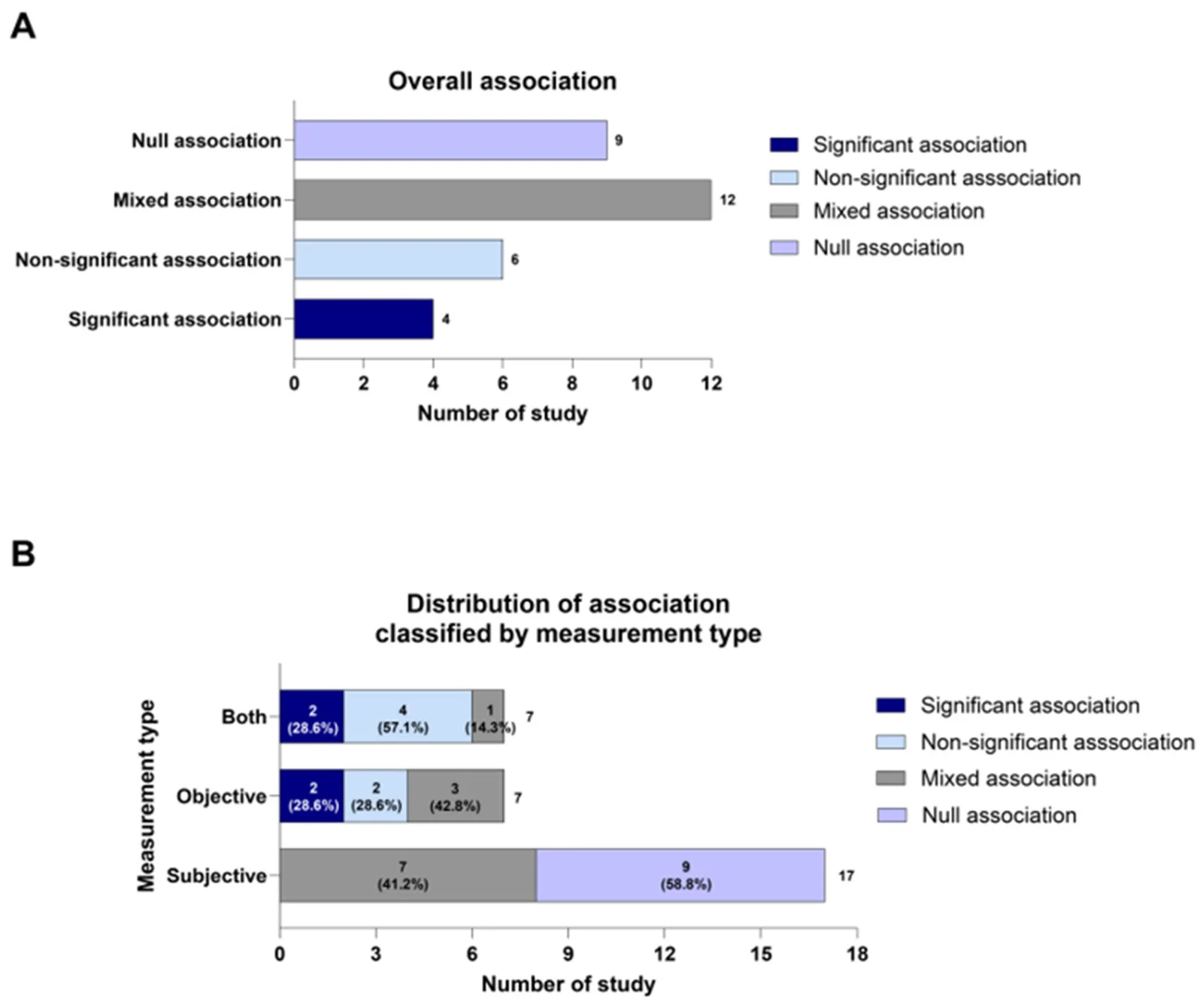
Bar charts summarizing cortisol–sleep associations in 31 reviewed studies, by significance and sleep measurement type. (**A**) The overall association was classified into 4 categories: significant association (*n* = 4), non-significant association (*n* = 6), mixed association (*n* = 12) and null association (*n* = 9). (**B**) Subgroup breakdown by measurement approach: subjective (*n* = 17; mixed association 41.2% and null association 58.2%), objective (*n* = 7; significant association 28.6%, non-significant association 28.6% and mixed association 42.8%), and combined methods (*n* = 7; significant association 28.6%, non-significant association 57.1% and mixed association 14.3%). Significant association refers to there being a statistically significant correlation; whereas non-significant association is when there is a correlation, but it is not statistically significant. In contrast, null association means there is no correlation at all. Mixed findings refer to studies reporting both statistically significant and non-significant associations between salivary cortisol and sleep quality, depending on sleep assessment methods, saliva sample collection timing, or outcome measures.

Figure 3 presents the findings on the association between salivary cortisol and sleep quality across the 31 studies included in this review. Overall, slightly more studies reported a significant association between salivary cortisol and sleep quality than those reporting no significant relationship (Figure 3A). Figure 3B further breaks down these findings by the type of sleep assessment used, including subjective, objective, and combined subjective and objective measures (both), highlighting how the reported associations varied across different measurement approaches.

Table 3 provides an overview of the characteristics of the included studies, grouped according to the sleep assessment method, salivary cortisol sampling protocol, and study population. It also summarizes the patterns of association between salivary cortisol and sleep quality observed within each category, allowing for comparison across different study designs and populations. Sleep assessment methods were classified as subjective only (questionnaires, rating scales, or sleep diaries), objective only (actigraphy or polysomnography), or combined subjective and objective assessments. Cortisol sampling protocols were classified according to the reported measurement approach; single-time-point sampling and multiple-time-point sampling, specifically the cortisol awakening response (CAR) and/or diurnal cortisol sampling. Healthy populations were distinguished from patient populations according to the study populations reported in Table 2. Descriptive patterns summarize the reported findings and do not represent pooled effect sizes or formal comparisons of statistical significance between methodological categories.

### 3.4. Discussion

Recently, the use of salivary cortisol has become more prevalent as a practical way to examine sleep-related physiology without invasive procedures, and the studies covered in this review provided supporting evidence for this approach. The increasing interest in salivary cortisol stems largely from its non-invasive nature and the feasibility of repeated sampling. Across the selected literature, associations have been reported between salivary cortisol, HPA-axis activity, and circadian regulation. As such, salivary cortisol may offer useful information about the physiological processes underlying sleep regulation, particularly those that fluctuate throughout the day, such as stress and circadian rhythms.

Compared with previous reviews of salivary cortisol in intervention-based and biobehavioral research [13,14], the present scoping review specifically maps the available evidence on the association between salivary cortisol and sleep quality. This review examines the reported associations in relation to sleep quality assessment and cortisol level, providing a more detailed overview of how these methodological differences may contribute to variations in the findings. This approach extends the scope of previous reviews by focusing specifically on sleep quality and highlights the importance of considering salivary cortisol levels and sleep assessment methods when interpreting the available evidence.

However, findings regarding the relationship between salivary cortisol and sleep quality were heterogeneous. There are several studies that observed changes in cortisol levels or the cortisol awakening response among individuals who have clinical conditions such as poor sleep quality, insomnia, woke frequently during the night, or experienced other ongoing sleep problems. The cortisol awakening response was repeatedly shown to be a point of sensitivity, with studies linking its disruption to irregularities in HPA-axis function [6,9] and changes in circadian regulation [19,20]. Despite these observations, there was limited consistency in the direction and magnitude of cortisol changes across studies.

In addition to cortisol awakening responses, many studies also investigated the diurnal cortisol slope, which is the gradual decrease in cortisol levels during the day. In normal physiological cases, cortisol levels reach their maximum shortly after awakening, decreasing throughout the day. A flatter diurnal cortisol slope, indicated by a slower and smaller decrease in cortisol levels, has been suggested to be associated with HPA-axis dysregulation and chronic physiological stress [1,25]. Several studies in this scoping review indicate that poor sleep quality, shorter sleep duration, and greater frequency of sleep disturbances correlated with changes in diurnal cortisol trends such as flatter cortisol slopes and decreased day-to-night differences [1,2,3,31]. These results could be explained by how disrupted sleep may interfere with and obstruct normal circadian regulation, as well as leading to prolonged activation of the HPA-axis. However, some studies reported different findings, showing no significant association between sleep quality measures and diurnal cortisol parameters [8,9]. This inconsistency in results suggests that the relationship between sleep quality and salivary cortisol is highly complex and requires critical and thorough methodological considerations.

Methodological differences between studies explain the contrast in findings between each study. Saliva collection protocols are substantially varying in terms of collection method, number and timing of samples, storage conditions, and laboratory cortisol detection assay [8,36]. Minor differences in these procedures can significantly affect cortisol values and complicate comparisons across studies. The timing of sampling is particularly important because cortisol rises rapidly after awakening and declines steadily thereafter. Studies that collected only a single sample at a fixed time point may have missed dynamic patterns relevant to sleep disruption [6]. However, as shown in Table 3, both significant and non-significant associations were reported among studies using multiple-time-point sampling. For example, associations between sleep parameters and diurnal cortisol patterns were reported in some studies [1,2,3], whereas others found no significant associations [8,9]. This suggests that multiple-time-point sampling alone does not guarantee the detection of significant associations, as the specific cortisol indices examined, sampling times, and sleep outcomes measured may also influence the findings.

The measurement method of sleep quality itself also contributed to variability in findings. Some studies relied solely on subjective tools such as the Pittsburgh Sleep Quality Index (PSQI), while others used objective methods such as actigraphy or polysomnography, or a combination of both methods. These approaches often yield different results, as perceived sleep quality does not always align with physiological sleep parameters [7,21]. Subjective measures can capture an individual’s experience of sleep and are practical for studies with large sample sizes, but they are susceptible to recall bias and emotional influences. Objective measures provide more direct physiological data but are more resource-intensive and may not fully reflect subjective sleep experience. The combination of these different measurement approaches across studies contributed to the scattered pattern of results observed in the literature.

When findings were considered according to sleep assessment methods, the direction of the observed associations varied across studies. Among studies relying exclusively on subjective sleep assessments (*n* = 17), some reported both significant and non-significant associations between salivary cortisol and sleep-related outcomes [19,20,23,24,26,31,34,36], nine studies found null association [6,9,17,22,25,32,33,35,38]. In contrast, among studies using objective assessment alone (*n* = 7), significant associations were reported for certain sleep parameters [3,7], while other studies reported no significant association [18,30]. Furthermore, three studies reported both significant and non-significant findings depending on the sleep parameters examined [1,21,27]. Among studies combining subjective and objective assessments (*n* = 7), significant associations were reported for selected sleep outcomes [2,37], whereas other studies found no significant association [5,8,10,29]. In addition, another study reported both significant and non-significant findings depending on the measurement methods and sleep parameters examined [28]. These descriptive patterns suggest that the choice of sleep assessment methods may contribute to the heterogeneity of findings. However, the small number of studies using objective assessment alone and the variation in sleep parameters and cortisol sampling protocols preclude firm conclusions regarding the superiority of any assessment method. These categories reflect reported association patterns rather than a formal comparison of statistical significance or effect sizes.

Moreover, one study found that variability in sleep timing and duration was significantly associated with diurnal cortisol slope, whereas average sleep duration and timing were not significantly associated with cortisol in adjusted models [1]. Similarly, another study reported significant association between sleep fragmentation and cortisol levels, while other measures of sleep continuity showed no significant association [21]. This indicates that different dimensions of sleep, such as duration, fragmentation, and variability, may not be equally associated with HPA-axis activity. Additionally, the presence of both significant and non-significant findings among studies using subjective assessments suggests that the use of self-reported measures alone may not explain the observed inconsistency [6,15,19,20]. Population characteristics may further contribute to these differences. Although altered cortisol patterns were reported in some clinical populations, associations with sleep outcomes were not consistent across studies, suggesting that underlying health conditions may influence cortisol secretion independent of sleep quality. Consequently, differences in participant characteristics, alongside cortisol sampling protocols and sleep assessment methods, make it difficult to isolate which methodological factor is most strongly associated with significant findings.

Overall, the relationship between salivary cortisol and sleep quality was inconsistent. A substantial number of studies, particularly those involving healthy volunteers or relying exclusively on self-reported sleep measures, found no clear association [17,28,29]. While salivary cortisol offers practical advantages over blood sampling, being non-invasive, well-tolerated, and suitable for repeated collection, the current evidence indicates that it cannot yet be confidently used as a standalone marker of sleep quality. Standardized collection protocols and greater integration of cortisol data with objective sleep measures would be needed to strengthen its utility.

This review has several limitations. Wide variation in participant populations, sleep assessment tools, and cortisol sampling methods made direct comparisons across studies difficult. The predominance of cross-sectional designs limited the ability to infer causal relationships. In addition, many included studies involved clinical populations (e.g., insomnia, sleep apnea, chronic illness) whose conditions can independently influence cortisol levels. Reporting quality was also inconsistent; several studies provided insufficient detail on saliva handling and storage procedures.

Additionally, no formal methodological quality or risk-of-bias assessment was conducted, as the primary objective of this scoping review was to map the available evidence rather than evaluate the effectiveness of specific interventions. Nevertheless, this limits the ability of this scoping review to assess the reliability of individual study findings and the overall strength of evidence. Small sample sizes were another important limitation, with several included studies involving fewer than 20 participants [20,22]. Such small samples may limit statistical power to detect associations between salivary cortisol and sleep quality and may also restrict the generalizability of the findings. Furthermore, potential publication bias cannot be excluded, as studies reporting statistically significant associations may be more likely to be published than those reporting null findings. The exclusion of gray literature may have further limited the range of available evidence and contributed to the underrepresentation of unpublished or non-significant findings.

Another limitation is the absence of a pooled quantitative synthesis of the associations between salivary cortisol and sleep outcomes. Although the included studies were summarized descriptively, substantial heterogeneity in the study designs, participant characteristics, sleep assessment methods, and cortisol sampling protocols limited the feasibility of deriving a meaningful pooled effect estimate. Specifically, differences in saliva collection timing complicated comparisons because cortisol concentrations follow a diurnal rhythm. For example, studies investigating the cortisol awakening response collected saliva following awakening [6,7], whereas other studies examined diurnal cortisol patterns using samples collected at multiple time points throughout the day [3,30]. Consequently, the cortisol parameters measured across these studies were not directly comparable. Differences in sleep outcome definitions and statistical reporting further limited the comparability of findings. Without pooled effect estimates, this review could not establish the overall magnitude or consistency of the association between salivary cortisol and sleep quality. Taken together, these limitations emphasize the need for cautious interpretations of the findings and limit the strength of the conclusions regarding the reliability of salivary cortisol as a biomarker of sleep quality.

In conclusion, this review indicates that the relationship between salivary cortisol and sleep is genuine but complex. Cortisol does not map directly onto sleep quality. Rather, it reflects broader aspects of stress-system functioning and circadian regulation, both of which interact with sleep in ways that vary across individuals, contexts, and research methods [6,9]. As shown in Table 3, many apparently conflicting findings become more understandable when methodological differences, particularly in cortisol sampling protocols and sleep assessment approaches, are taken into account [1,7]. Advancing the field will require greater standardization of methods rather than simply conducting more studies using existing approaches.

## 4. Conclusions

Although the current evidence is insufficient to support the use of salivary cortisol as a reliable standalone biomarker of sleep quality, the existing findings suggest that it may have potential in this context. Further research is needed to establish its reliability and validity as an indicator of sleep quality. Even though there are many studies that investigate associations between sleep outcomes and cortisol levels, the results of such studies were very inconsistent across populations, study designs, and measurement approaches. Its utility lies in its ability to reflect broader physiological processes, and its interpretation should be contextualized within a multidimensional framework of sleep and stress. The most prominent source of variability across the studies was methodological heterogeneity. This could be due to differences in saliva collection timing, sampling frequency, cortisol awakening response protocols, storage conditions, analytical assays, and sleep assessment methods. Additionally, there are certain extraneous variables such as psychological stress, age, health, use of medication, and environmental influences that may also affect cortisol secretion independently of sleep, obstructing the interpretation of findings. Such studies that employ methodologies like repeated sampling protocols, such as diurnal cortisol profiles and multiple cortisol awakening response measurements, generally demonstrated stronger and more consistent associations than studies relying on single-time-point samples. Consequent research should emphasize the use of standardized cortisol collection methods, validated and comprehensive sleep assessment tools, and comprehensive control of potential confounding variables. Taken together, this scoping review suggests that salivary cortisol should be interpreted and used within a multidimensional framework of sleep and stress, specifically by being integrated as part of a broader assessment strategy instead of an independent measure of sleep quality.

## 5. Limitations

The included studies were characterized by substantial heterogeneity in design, population, and measurement approaches, limiting comparability. The reliance on subjective sleep measures in many studies introduces potential bias, and the variability in cortisol protocols further complicates interpretation.

## Figures and Tables

**Table 1:** Search strategies in databases.

Database	Number of Records	Search String
Scopus	431	TITLE-ABS-KEY ((“Cortisol” OR “Salivary Cortisol”) AND (“Sleep” OR “Sleep Quality”) AND (“Biomarker” OR “Biosensor”))
Medline/PubMed	454	((Cortisol[Title/Abstract]) OR (Salivary Cortisol[Title/Abstract])) AND ((Sleep[Title/Abstract]) OR (Sleep Quality[Title/Abstract])) AND ((Biomarker[Title/Abstract]) OR (Biosensor[Title/Abstract]))
CENTRAL	182	((Cortisol) OR (Salivary Cortisol)) AND ((Sleep) OR (Sleep Quality)) AND ((Biomarker) OR (Biosensor))

**Table 2:** Summary of included studies.

Author(s) and Year	Country	Aims of the Study	Population	Study Design	Sleep Measurements	Cortisol Sampling	Key Finding
Varkevisser et al. (2005) [18]	Netherlands	This study determines whether chronic insomnia is associated with altered physiological markers of arousal (cortisol, body temperature, heart rate) across 24 h under constant-routine conditions.	24 subjects (11 patients with chronic insomnia and 13 healthy matched controls)	Case–control study	Objective (PSG)	Salivette^®^ −20 °C Diurnal 9 samples over 24 h ELISA	The results indicated that no statistically significant associations were found between insomniacs and controls in cortisol levels.
Brand et al. (2012) [19]	Switzerland	This study investigates the impact of long- and short-term meditation experience on the HPA system and sleep.	20 participants, consisted of healthy adults	Cross-sectional study	Subjective (PSQI)	Saliva swab –20 °C CAR Awakening RIA	Lower CAR was associated with better sleep quality. Long-term meditators showed progressively lower CAR and had significantly better sleep than novices, while an 8-week MBSR course in novices reduced CAR and improved sleep, with post-course higher CAR correlating with poorer sleep.
Nakano et al. (2012) [20]	Japan	This study evaluates the effectiveness of pyrroloquinoline quinone (PQQ) on stress, fatigue, quality of life, and sleep in adult workers.	17 subjects with no specification	Non-randomized experimental	Subjective (PSQI, AIS)	Saliva swab Home refrigerator (4 °C) CAR Awakening ELISA	CAR changes correlated with PSQI sleep score changes (R = −0.55, *n* = 7) with higher CAR results in better sleep quality, biomarker support for improved sleep quality.
Casida et al. (2013) [21]	USA	This study describes sleep patterns and biomarkers of stress/inflammation post-CPB, determines the changes over 4 days, and explores the relationships between sleep characteristics and these biomarkers.	20 participants, consisting of adult males and females aged 18 years or older, and scheduled for first-time elective cardiac surgery using cardiopulmonary bypass (CPB)	Cohort study	Objective (Actigraphy)	Saliva swab −56.7 °C Diurnal Morning ELISA	High sleep fragmentation (more disrupted, lower-quality sleep) was strongly associated with higher cortisol levels. Other sleep continuity measures (total sleep time, efficiency, WASO, onset latency) showed no significant association with cortisol.
Ohiro et al. (2013) [22]	Japan	This study evaluates the impact of deodorized garlic extract on subjective, sleep quality, negative mental mood, and biological stress markers in workers.	6 men, 11 women, the population consisted of workers experiencing high levels of sleep disturbance and fatigue prior to the intervention	Open-label design	Subjective (PSQI, OSA-MA)	Collection method not stated Storage condition not stated CAR Awakening ELISA	CAR did not change significantly after 8 weeks on weekdays or holiday; sleep parameters were not supported by a CAR biomarker change in this study.
Vargas et al. (2013) [23]	USA	This study aimed to differentiate the effects of daily life hassles, anticipatory stress, and subjectively reported sleep on CAR.	58 young adults	Cross-sectional study	Subjective (PSQI)	Salivette® −20 °C CAR Awakening RIA	Results showed that total sleep time (TST) was the strongest predictor of awakening cortisol and CAR. Lower TST was associated with lower cortisol at awakening and greater post awakening cortisol activation hassles did not have a significant effect on awakening cortisol.
Pesonen et al. (2014) [3]	Finland	This study examines the associations between objectively measured sleep duration/quality and diurnal cortisol patterns and determines if sleep is associated with HPA-axis feedback inhibition.	305 adolescents	Cohort study	Objective (Actigraphy)	Salivette® −20 °C CAR, diurnal Multiple samples DELFIA	In adolescent boys, short sleep duration was associated with higher cortisol upon awakening, and lower sleep quality showed a trend toward lower CAR.
Lopez-Jornet et al. (2015) [24]	Spain	This study evaluates psychological features, sleep, and salivary biomarkers in OLP patients compared with controls.	65 participants, consisting of men and women aged ≥18 years, with the presence of oral lichen planus (OLP) affecting the oral mucosa	Cross-sectional study	Subjective (PSQI)	Passive drool −80 °C Single sample Noon Chemiluminescent immunoassay	Cortisol appeared to track with sleep quality at the group level, with the OLP patients having both significantly higher cortisol and significantly worse PSQI scores than controls, suggesting the two rise together with disease status.
Sladek & Doane (2015) [8]	USA	This study examines within-person associations between daily social connection, objective sleep duration/quality, and diurnal cortisol patterns the following day.	71 first-year college students	Longitudinal naturalistic field study utilizing a daily diary approach and ecological momentary assessment	Subjective (Sleep diary) and Objective (Actigraphy)	Passive drool −20 °C Multiple sample 5 time points per day TR-FIA	No significant association was found between any objective sleep parameter (time in bed, time asleep, sleep latency) and any cortisol index (waking levels, CAR, diurnal slope).
Slatcher et al. (2015) [25]	China	This study investigates the associations between positive and negative coping strategies and diurnal cortisol among children affected by parental HIV/AIDS.	335 boys and 310 girls aged between 6 and 17 years old	Cross-sectional study	Subjective (Likert sleep)	Salivette® Room temperature, then refrigerated Diurnal Awakening, Evening, Bedtime Chemiluminescent immunoassay	Sleep quality, both as a daily measure and as a 3-day average, showed no association with morning cortisol levels or the diurnal cortisol slope in this sample of children affected by parental HIV/AIDS.
Vargas et al. (2016) [17]	USA	This study investigates whether the link between depressive symptomatology and CAR varies as a function of sleep patterns and gender.	58 healthy young adults	Cross-sectional study	Subjective (Sleep diary)	Salivette® Storage condition not stated Single sample Morning ELISA	There was no cortisol—OSA Association.
Bei et al. (2017) [1]	USA	This study assesses associations between objectively measured sleep IIV and cortisol diurnal rhythm, and multisystem physiological dysregulation (AL).	436 adults drawn from the MIDUS 2 biomarker Sub-study and were community-dwelling adults who were part of a nationally representative sample in the United States	Cohort study	Objective (Actigraphy)	Salivette® Storage condition not stated Diurnal Multiple samples Cortisol assay not stated	The variables sleep timing (Rise Time and Bedtime) and duration (Total Sleep Time) were significantly associated with diurnal cortisol slope. In adjusted models, mean sleep variables were not associated with cortisol, suggesting that variability is a stronger predictor than average sleep duration for this outcome.
Lin et al. (2017) [26]	China	This study aimed to determine whether salivary cortisol concentrations predict the efficacy of a sleep promoting treatment in children with postural tachycardia syndrome (POTS) and to compare salivary cortisol levels and sleep quality between children with POTS and healthy controls.	60 subjects (40 children with postural) tachycardia syndrome (POTS) and 20 healthy controls	Cohort study	Subjective (PSQI)	Salivette® −80 °C CAR, Diurnal 6 time points per day ELISA	Results indicated that higher cortisol predicted response to treatment, and CAR positively correlated with both PSQI scores and clinical symptom scores.
Engert et al. (2018) [9]	Germany	This study examines the multidimensional structure of the stress response and its relationship to health and sleep outcomes by integrating salivary cortisol with behavioral and self-reported measures.	328 healthy participants	Cohort study	Subjective (PSQI, sleep diary)	Passive drool −30 °C CAR, Diurnal Awakening, Noon, Evening TR-FIA	Subjective sleep quality showed no association with cortisol in this large, healthy sample. However, the awakening time could predict participants’ awakening cortisol, with earlier risers showing higher levels of both.
Hernández et al. (2018) [27]	USA	This study examines associations between morning salivary cortisol summary parameters and behavioral correlates such as stress, sleep, fatigue, and physiological variables in elite military men.	58 active personnel, with a compliant subgroup of 29 participants used for key cortisol analyses	Cohort study	Objective (Actigraphy)	Saliva swab −80 °C CAR Awakening ELISA	The result indicated that cortisol may not be a reliable standalone biomarker of sleep quality in elite military personnel.
Mikoteit et al. (2018) [28]	Switzerland	This study investigates the associations of brain-derived neurotrophic factor (sBDNF) with subjective and objective sleep, dimensions of psychopathology, and cortisol secretion.	Insomnia Group (IG): 31 male, 29 female. Control group (CG): 13 male, 17 female	Case–control study	Both Subjective (ISI) and Objective (PSG)	Salivette® −20 °C CAR Awakening RIA	Worse subjective sleep (higher insomnia severity) was linked to lower morning cortisol output, not elevated cortisol, contradict to the hyperarousal hypothesis. Objective sleep continuity was largely unrelated to cortisol, only sleep architecture (REM%, NREM S2%) showed possible associations.
Haraki et al. (2019) [29]	Japan	This study investigates objective and subjective characteristics of sleep, psychosomatic, and physiologic variables in young subjects with different frequencies of RMMA.	54 healthy young adults	Cross-sectional study	Subjective (5-grade Likert scale) and Objective (PSG)	Salivette® −20 °C Diurnal Awakening, Evening ELISA	There was no association between sleep and cortisol level.
Kramer et al. (2019) [6]	Germany	This study investigates whether anticipated stress for the next day (assessed in the evening) predicts the post-awakening cortisol increase the following morning.	42 healthy participants between 18 and 40 years old	Longitudinal EMA	Subjective (Self-report)	Passive drool −80 °C CAR Awakening ELISA	Sleep quality showed no significant association with the CAR at either the within-person or between-person level.
Yan et al. (2019) [30]	China	This study assesses the utility of salivary cortisol and alpha-amylase levels in assessing OSA severity and concomitant hypertension.	58 subjects which had varying severity of OSA	Cross-sectional study	Objective (PSG)	Saliva swab −80 °C CAR, Diurnal 6 time points per day ELISA	Salivary cortisol was not associated with OSA severity but was significantly higher in patients with hypertension, regardless of OSA status.
Huang et al. (2020) [31]	Taiwan	This study investigates the relationship between diurnal cortisol profiles and sleep quality in patients with hepatocellular carcinoma and compares these patterns with healthy controls.	108 participants (75 patients with hepatocellular carcinoma and 33 healthy controls)	Cohort study	Subjective (PSQI)	Salivette −70 °C Diurnal Multiple sample ELISA	The association between cortisol and poor sleep quality was a weak negative correlation, indicating a small effect size despite statistical significance (OR = 1.00007).
LaVoy et al. (2020) [2]	USA	This study explores bidirectional relationships between objective sleep variables and salivary biomarkers of stress and immunity in youth.	55 children and adolescents aged 8–16 years recruited from Houston, Texas	Cohort study	Subjective (Sleep Diary) and Objective (Actigraphy)	Passive drool −80 °C Diurnal Multiple samples Competitive immunoassay	More time spent awake during the night was associated with higher morning cortisol, and higher morning cortisol predicted lower sleep efficiency and more wake time.
Anderson et al. (2021) [7]	USA	This study assesses CAR association and objectively measures sleep duration and physical activity.	58 healthy young adults	Cohort study	Objective (Actigraphy)	Passive drool −20 °C CAR Awakening DELFIA	There was a significant interaction between prior-day sleep duration, CAR level, and physical activity.
Lopresti et al. (2021) [32]	Australia	This study examines the effect of Bacopa monnieri (Bacognize^®^) on stress, fatigue, quality of life, and sleep in adults with self-reported poor sleep.	100 adults, males and females with poor sleep aged 18 to 70, BMI between 20 and 30, and usual bedtime between 9:00 p.m. and 12:00 a.m.	Randomized controlled trial	Subjective (PSD, BIS)	Passive drool Storage condition not stated CAR, Diurnal Awakening, evening ELISA	Cortisol difference did not associate with better sleep outcomes (BIS, FOSQ-10, PSD changes were similar between groups).
Liu et al. (2022) [33]	USA	This study examines whether daily cortisol total output mediates the association between daily sleep and effect on high-stress and low-stress days.	173 dementia family caregivers	Cohort study	Subjective (Sleep diary)	Collection method not stated Storage condition not stated Diurnal Multiple samples Cortisol assay not stated	There was no sleep–cortisol association.
Cho et al. (2023) [5]	Korea	This study investigates time-dependent changes in salivary hormones, their relationship with the sleep–wake cycle, and correlations with BPSD and behavioral factors.	172 older adults (over 65 years old) with dementia	Cohort study	Subjective (Sleep diary) and Objective (Actigraphy)	Passive drool –80 °C Diurnal Multiple Samples ELISA	There is no association between sleep and cortisol level.
So et al. (2023) [10]	USA	This study investigates putative mediators (waking cortisol and nighttime arousal) and moderators (CSA characteristics) of associations between PTSS and sleep patterns among women with CSA history.	64 women between 18 and 40 years old, self-reported history of at least one childhood sexual abuse (CSA) event before age 12	Cross-sectional study	Subjective (PSQI, sleep diary) and Objective (Actigraphy)	Passive drool −80 °C CAR Awakening ELISA	There was no sleep–cortisol association
Ballestar-Tarín et al. (2024) [34]	Spain	This study analyzes the relationship between salivary cortisol and BDNF levels with sleep quality and psychological alterations in university students.	72 nursing degree students from the University of Valencia (Spain)	Cross-sectional study	Subjective (PSQI)	Salivette −80 °C Single sample Noon ELISA	Higher morning salivary cortisol levels were significantly associated with poorer sleep quality (higher PSQI scores)
Mateos-Olivares et al. (2024) [35]	Spain	This study evaluates the utility of salivary biomarkers as non-invasive biochemical markers for sleep deprivation and anxiety in retinitis pigmentosa (RP) patients.	112 participants, aged 18 to 60 years, with healthy conditions and diagnosed with retinitis pigmentosa	Cross-sectional study	Subjective (PSQI)	Passive drool −20 °C Diurnal Noon ELISA	Salivary cortisol showed no significant difference between normal sleepers and poor sleepers in RP patients, despite having different sleep quality. Thus, cortisol cannot accurately predict sleep quality.
Pachimsawat et al. (2024) [36]	Thailand	This study examines the effect of acute exogenous melatonin administration on salivary cortisol and alpha amylase as representatives of the HPA axis and the sympathetic nervous system, respectively.	64 healthy volunteers, at least 18 years old	Randomized controlled trial	Subjective (PSQI, Visual Analog Scale, Sleep diary)	Passive drool −20 °C then −80 °C Diurnal Multiple samples Competitive Immunoassay	Participants with poor sleep quality (PSQI > 5) had a more increase in salivary cortisol levels following melatonin consumption compared with participants with normal baseline sleep quality.
Lee et al. (2025) [37]	Taiwan	This study determines whether eight weeks of supplementation with heat-treated *Limosilactobacillus fermentum* PS150 could improve sleep quality in adults with poor subjective sleep and explores associated changes in sleep-related biomarkers across blood, saliva, and urine.	84 participants, aged 20–60 years with poor sleep quality	Randomized controlled trial	Subjective (PSQI, ISI) and Objective (Wearable device)	Collection method not stated Storage condition not stated Diurnal Multiple samples ECLIA	Cortisol level decreases with mean subjective sleep outcome improved.
Watanabe et al. (2025) [38]	Japan	This study determines if *Lactiplantibacillus plantarum* SNK12 intake helps improve sleep quality and alters stress-related immune and neuroendocrine biomarkers in adults.	56 healthy Japanese adults reporting morning fatigue or dissatisfaction with sleep quality in daily life	Randomized controlled trial	Subjective (OSA-MA)	Salivette −80 °C Single sample Morning ELISA	Salivary cortisol was significantly lower with L. plantarum SNK 12 than placebo at week 4, and this decrease aligned directionally with improved sleep scores (Sleepiness on Rising, Initiation/Maintenance of Sleep), supporting a cortisol–sleep link via HPA-axis modulation.

**Table 3:** Methodological characteristics and reported patterns of association between salivary cortisol and sleep quality.

Feature	Category	Number of Studies	Descriptive Pattern
Sleep Measurement	Subjective	17	Both significant and non-significant associations and null findings were reported; results varied across self-reported sleep outcomes.
	Objective	7	Significant associations were reported for specific sleep parameters such as variability in sleep timing and duration and sleep fragmentation, whereas other parameters including mean sleep measures and certain measures of sleep continuity showed no significant associations.
	Both Subjective and Objective	7	Findings were inconsistent across studies and, in some cases, differed between subjective and objective sleep parameters.
Cortisol Protocol	Single-time-point sampling	4	A single cortisol measurement does not characterize the cortisol awakening response (CAR), or diurnal rhythm findings were not consistently associated with sleep outcomes.
	Multiple-time-point sampling (CAR/diurnal)	27	Both significant and non-significant associations were reported, indicating that multiple-time-point sampling did not consistently identify relationships with sleep outcomes.
Population	Healthy populations	22	Both significant and non-significant associations and null findings were reported across populations without a defined clinical condition.
	Patient populations	9	Altered cortisol patterns were reported in some patient groups, although associations with sleep outcomes were not consistent.

## Data Availability

No new primary data were created or analyzed in this study. Data sharing is not applicable to this article.

## Abbreviations

The following abbreviations are used in this manuscript:

BPSD	Behavioral and psychological symptoms of dementia
CAR	Cortisol Awakening Response
ECLIA	Electrochemiluminescence Immunoassay
PSQI	Pittsburgh Sleep Quality Index
OSA	Obstructive Sleep Apnea
PSD	Pittsburgh Sleep Diary
OLP	Oral lichen planus
PSG	Polysomnography
RIA	Radioimmunoassay
ELISA	Enzyme-Linked Immunosorbent Assay
ISI	Insomnia Severity Index
EMA	Ecological Momentary Assessment
BIS	Barratt Impulsiveness Scale
TR-FIA	Solid phase time-resolved fluorescence immunoassay with fluorometric endpoint detection
MIDUS	Midlife in the United States Study
DELFIA	Dissociation-Enhanced Lanthanide Fluorescent Immunoassay
POTS	Postural tachycardia syndrome
BMI	Body Mass Index
FOSQ-10	Functional Outcomes of Sleep Questionnaire
REM	Rapid eye movement
NREM S2	Non-rapid eye moment stage 2
PTSS	Post-Traumatic Stress Syndrome
CSA	Childhood Sexual Abuse
